# Characterizing the Antihyperglycemic Activity and Underlying Mechanisms of the Aqueous Extract of the Leaves from *Ficus carica* L.

**DOI:** 10.3390/molecules31132207

**Published:** 2026-06-23

**Authors:** Fernando Calzada, Jesica Ramírez-Santos, Hannia Pérez-Álvarez, Miguel Valdes, Elizabeth Barbosa, Claudia Velázquez

**Affiliations:** 1 Unidad de Investigación Médica en Farmacología, UMAE Hospital de Especialidades, 2° Piso CORSE, Centro Médico Nacional Siglo XXI, Instituto Mexicano del Seguro Social, Av. Cuauhtémoc 330, Col. Doctores, Mexico City 06725, Mexico; hannyperezalvarez@gmail.com (H.P.-Á.); valdesguevaramiguel@gmail.com (M.V.); 2 Instituto Politécnico Nacional, Sección de Estudios de Posgrado e Investigación, Escuela Superior de Medicina, Plan de San Luis y Salvador Díaz Mirón S/N, Col. Casco de Santo Tomás, Miguel Hidalgo, Mexico City 11340, Mexico; rebc78@yahoo.com.mx; 3 Facultad de Medicina, Universidad Veracruzana, Agustín de Iturbide S/N, Zona Centro, Veracruz 91700, Mexico; 4 Laboratorio de Inmunología, Departamento de Sistemas Biológicos, Universidad Autónoma Metropolitana Unidad Xochimilco, Calz. del Hueso 1100, Coapa, Villa Quietud, Coyoacán, Mexico City 04960, Mexico; 5 Área Académica de Farmacia, Instituto de Ciencias de la Salud, Universidad Autónoma del Estado de Hidalgo, Circuito ex Hacienda La Concepción S/N, Carretera Pachuca-Atocpan, San Agustin Tlaxcala 42076, Mexico; cvg09@yahoo.com

**Keywords:** *Ficus carica*, type 2 diabetes mellitus, antihyperglycemic activity, *a*-glucosidase inhibitor, lipid profile, narcissin, nicotiflorin, *β*-sitosterol

## Abstract

*Ficus carica* L. is traditionally used for diabetes management. This study evaluated the antihyperglycemic activity, safety, possible mechanisms, and phytochemical composition of its aqueous leaf extract (EAcFc). EAcFC activity was evaluated in streptozotocin–nicotinamide-induced type 2 diabetic (ST2D) mice under acute and subchronic conditions. EAcFc showed low acute toxicity (LD_50_ > 3000 mg/kg). Acute and subchronic oral administration of EAcFc (300 mg/kg) significantly reduced blood glucose levels in ST2D mice. Although sustained HbA1c reduction was not observed, EAcFc improved lipid profiles, notably reducing triglyceride concentrations in ST2D males (from 156 ± 19.4 to 89.7 ± 3.3 mg/dL at week 4) and females (from 138 ± 2.0 to 77 ± 16.0 mg/dL at week 4). In oral sucrose and lactose tolerance tests (3 g/kg load), EAcFc (300 mg/kg) significantly attenuated postprandial hyperglycemia at 30, 60, and 120 min, an effect comparable to acarbose (50 mg/kg). No significant activity was observed during the oral glucose tolerance test (1.5 mg/kg load), suggesting the effect is not mediated by SGLT-1 inhibition. Preparative TLC and NMR analysis identified narcissin, nicotiflorin, and β-sitosterol. Thus, EAcFc possesses antihyperglycemic and lipid-modulating properties partially associated with α-glucosidase inhibition and bioactive flavonoids and phytosterol.

## 1. Introduction

Diabetes mellitus (DM) is a chronic metabolic disorder characterized by the presence of persistent hyperglycemia, caused by defects in insulin secretion, peripheral action, or both mechanisms simultaneously. This condition alters the metabolism of carbohydrates, lipids, and proteins, and is associated with progressive damage to multiple organs and systems, especially the cardiovascular, renal, nervous, and visual systems [1]. Among its classification, the most prevalent is type 2 diabetes mellitus (DM2), which represents the most frequent form and is related to insulin resistance and progressive functional deterioration of pancreatic β cells [2]. Worldwide, diabetes is one of the main public health challenges. Estimates for 2024 indicate that more than 800 million adults are living with this disease, with a sustained increase in its prevalence in recent decades, especially in low- and middle-income countries [3]. This increase is mainly due to changes in lifestyles, including high-calorie diets, decreased physical activity, and the increased prevalence of obesity. In Mexico, diabetes represents one of the main causes of morbidity and mortality, with an estimated prevalence by 2024 of approximately 16% in the adult population, placing the country among the countries with the highest burden of diseases worldwide [4,5]. The etiology of DM is multifactorial and results from the interaction between genetic, environmental and behavioral factors. Among the main risk factors are overweight and obesity, particularly the accumulation of visceral fat, sedentary lifestyle, inadequate diet and aging. These factors contribute to the development of insulin resistance, a condition in which peripheral tissues have a decreased response to this hormone, which leads to an initial compensation through hyperinsulinemia and over time this adaptation is insufficient due to the progressive deterioration of the function of pancreatic β cells, which leads to the establishment of chronic hyperglycemia and the development of the disease [6,7]. From a pathophysiological point of view, chronic hyperglycemia induces a series of biochemical alterations, including increased oxidative stress, activation of inflammatory pathways, and the formation of advanced glycation end products (EFAs), which play a central role in the development of associated complications [7,8]. These complications are classified as microvascular, such as diabetic nephropathy, retinopathy, and neuropathy, and macrovascular, which include cardiovascular and cerebrovascular diseases, the main causes of death in patients with diabetes [9]. In addition, there is a close relationship between DM2 and alterations in the lipid profile, mainly characterized by an increase in triglycerides (TGs), an increase in low-density lipoproteins (LDLs), especially small and dense particles, and a decrease in high-density lipoproteins (HDLs). These alterations are a consequence of insulin resistance, which increases lipolysis in adipose tissue, increases the release of free fatty acids to the liver, and favors hepatic overproduction of LDLs, contributing to metabolic deterioration and increased cardiovascular risk [10,11].

The pharmacological treatment of DM2 includes various oral medications, including biguanides such as metformin, thiazolidinediones such as pioglitazone and rosiglitazone, and sulfonylureas such as glibenclamide, as well as dipeptidyl peptidase-4 (iDPP-4) inhibitors and sodium–glucose cotransporter type 2 (SGLT2) inhibitors, such as canagliflozin, which act through different mechanisms to improve glycemic control [12]. However, several of these drugs may cause adverse effects or hypersensitivity reactions in some patients. Among the most frequent side effects are hypoglycemia, weight gain, gastrointestinal disturbances and pancreatitis [12,13]. Likewise, the use of thiazolidinediones has decreased in recent years due to the appearance of undesirable effects, such as fluid retention, edema, congestive heart failure, bone fractures, and possible association with certain types of cancer [14,15]. Due to these limitations, there has been increased interest in developing new therapeutic alternatives for DM2, focused on improving glycemic control, delaying complications and reducing adverse effects. In this context, medicinal plants have gained relevance as a possible complementary therapeutic option [16]. The World Health Organization has reported the existence of approximately 21,000 medicinal plants worldwide, of which about 400 have been identified as having therapeutic potential for the treatment of diabetes [17].

One of the botanical families to which antihyperglycemic properties are attributed is the Moraceae family, within which several species of the genus Ficus have been studied for their therapeutic potential in the management of DM. Among them, *Ficus religiosa*, *Ficus benghalensis*, *Ficus racemosa*, *Ficus glumosa*, *Ficus deltoidea* and *Ficus carica* have shown antihyperglycemic, antioxidant, antimicrobial, antifungal, anti-inflammatory and anticancer activity in various experimental models in vitro and in vivo; particularly the antihyperglycemic activity has been demonstrated by mechanisms related to the stimulation of the secretion of insulin, inhibition of digestive enzymes, and improvement in peripheral glucose uptake [18,19]. One of the species with the greatest economic, food, and medicinal importance is *Ficus carica* L., (*F. carica*) commonly known as fig tree; it is a fruit tree distributed in temperate and subtropical regions of Asia, Europe, Africa, South America, and the Mediterranean region [20,21]. In traditional medicine, different parts of this species, particularly its leaves, fruits, and latex, have been employed for the treatment of various conditions, such as gastrointestinal disorders, inflammation, infections, cardiovascular disease, and DM [18,19,20,21,22]. Several studies have reported that *F. carica* leaves exhibit multiple biological activities, which are antioxidant, anti-inflammatory, hepatoprotective, antimicrobial, antinociceptive, and antihyperglycemic [22,23]. Phytochemical studies of the leaves have identified flavonoids, anthocyanins, phenolic compounds, coumarins, terpenes, alkaloids, and organic acids, with compounds such as rutin, quercetin, chlorogenic acid, and ficusin being some of the main bioactive metabolites associated with their pharmacological effects [20,21,22,23,24]. Preclinical studies have shown that aqueous, ethanolic and methanolic extracts of *F. carica* leaves possess antihyperglycemic activity in animal models with streptozotocin- or alloxan-induced diabetes, suggesting their possible usefulness as adjunctive therapy in glycemic control [18,24,25]. The antihyperglycemic activity of *F. carica* leaves could be attributed to several mechanisms of action, such as its antioxidant capacity, associated with phenolic compounds and flavonoids such as quercetin, rutin, and luteolin, which reduce oxidative stress and protect the functionality of pancreatic β cells. In addition, residual insulin secretion improves peripheral glucose uptake and increases insulin sensitivity in target tissues. In a complementary manner, it has been proposed that some of its metabolites could inhibit digestive enzymes such as α-amylase and α-glucosidase, decreasing intestinal absorption of carbohydrates and postprandial hyperglycemia [18,20,21,22,23,24,25]. These findings support the fact that *F. carica* represents a promising alternative for the development of new therapeutic strategies in DM.

Despite the ethnopharmacological and preclinical background supporting the antihyperglycemic potential of *F. carica* leaves, there is a scarcity of robust evidence regarding the efficacy and safety of its traditional aqueous extracts in established T2DM models [18,23]. Addressing this gap holds significant practical interest. Scientifically validating the biological activity of its traditional preparation provides a pharmacological basis for its potential use as an accessible and low-cost complementary therapy for glycemic control [16]. Additionally, correlating its pharmacological effects with specific bioactive metabolites supports the future standardization of phytomedicines and the potential development of novel antidiabetic agents with fewer adverse effects [19]. Therefore, the present study aimed to evaluate the antihyperglycemic activity of the aqueous extract of *F. carica* leaves (EAcFc) in acute models of streptozotocin-induced DM2(ST2D). Subsequently, subchronic studies were carried out to determine its effect on sustained glycemic control and on associated metabolic parameters, including glycosylated hemoglobin (HbA1c) and lipid profile. In addition, to explore its possible mechanism of action, its inhibitory capacity on the enzyme α-glucosidase and on the sodium–glucose cotransporter type 2 (SGLT2) was evaluated using oral sucrose, lactose, and glucose tolerance assays (OST, OLT, and OGT, respectively). Finally, the isolation and identification of some of the metabolites present in the aqueous extract was carried out to associate its phytochemical composition with the pharmacological activity observed.

## 2. Results

### 2.1. Acute Oral Toxicity

The acute oral toxicity of the aqueous extract of the leaves from *F. carica* (EAcFc) was determined by the acute toxicity class method, following the guidelines established in guideline 423 of the Organization for Economic Cooperation and Development (OECD) for the evaluation of compounds with potential exposure in humans [26]. The results obtained showed that the EAcFc did not induce mortality or cause obvious behavioral changes in the experimental animals throughout the observation period. Also, at the end of the study (day 14), macroscopic inspection of the internal organs revealed no apparent alterations or tissue lesions, and no significant decrease in the body weight of the treated organisms was recorded. Based on these findings, EAcFc was classified within category 5 of acute toxicity (LD_50_ > 3000 mg/kg), indicating low toxicity and suggesting a wide margin of safety for its potential application in humans.

### 2.2. Acute Antihyperglycemic Evaluation of Aqueous Extract of Leaves from F. carica in Female and Male Mice with Streptozotocin-Induced Type 2 Diabetes Mellitus

**Figure 1:**
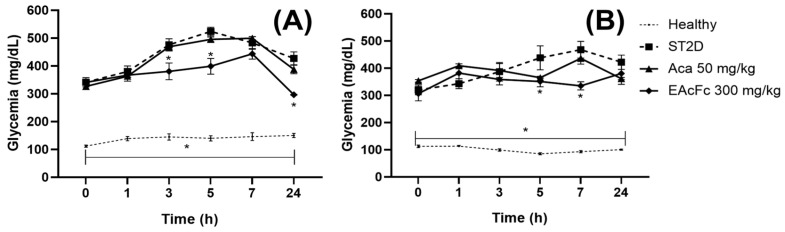
Results of the acute antihyperglycemic evaluation, (**A**) male mice and (**B**) female mice. Data are expressed as mean ± SEM (*n* = 6). Statistical analysis was performed using two-way ANOVA followed by Tukey’s multiple comparisons test. Differences were considered statistically significant when * *p* < 0.05 vs. ST2D comparing the treated groups versus the untreated diabetic control group (ST2D) at the same evaluated time point. ST2D: type 2 diabetic group induced by streptozotocin; EAcFc: aqueous extract of *F. carica* leaves; Aca: acarbose.

An acute study was conducted to evaluate the effect of aqueous extract of *Ficus carica* leaves (EAcFc) on blood glucose levels in mice of both sexes. The results obtained in the model of type 2 diabetes mellitus induced by streptozotocin (ST2D), as well as in normoglycemic (healthy) animals, are presented in Figure 1. In both sexes, the diabetic control group (ST2D) maintained elevated glucose concentrations throughout the experimental period (0–24 h) compared to the healthy group. In male mice (Figure 1A), the administration of EAcFc showed a modulating effect on glycemia, characterized by a tendency to decrease after 3 h and a more marked reduction towards 24 h, where statistically significant differences were observed with respect to the ST2D group. In females (Figure 1B), the antihyperglycemic effect was less pronounced, with significant reductions only observed in intermediate times (5 and 7 h) compared to the untreated diabetic group. When compared with the reference drug, acarbose (Aca), EAcFc showed greater antihyperglycemic activity in males, while in females it presented a comparable effect during the first hours of the experiment and after more than 7 h. Taken together, these results indicate that EAcFc has an acute antihyperglycemic effect in both sexes, with a time-dependent response and with differences in the magnitude of the effect between males and females.

### 2.3. Subchronic Evaluation of Aqueous Extract of Leaves from F. carica in Female and Male Mice with Streptozotocin-Induced Type 2 Diabetes Mellitus

**Figure 2:**
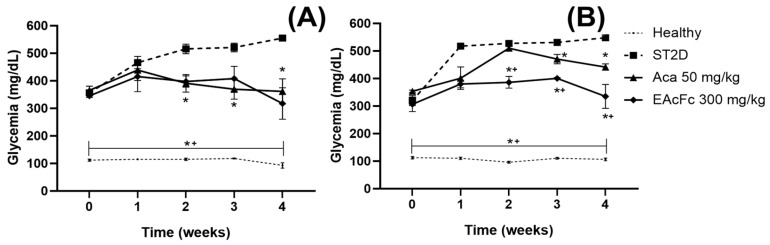
Results of the subchronic antihyperglycemic evaluation: (**A**) male mice and (**B**) females. Data are expressed as means ± SEM (*n =* 6). Statistical analysis was performed using two-way ANOVA followed by Tukey’s multiple comparisons test. Differences were considered statistically significant when * *p <* 0.05 comparing the indicated treatment versus the untreated diabetic control group (ST2D), and + *p <* 0.05 comparing the EAcFc group versus the reference drug (Aca) at the same evaluated week. ST2D: type 2 diabetic group induced by streptozotocin; EAcFc: aqueous extract of *F. carica* leaves; Aca: acarbose.

In order to evaluate the subchronic antihyperglycemic effect of EAcFc, the treatment was administered for four weeks in ST2D. The results obtained are shown in Figure 2. In both sexes, the untreated diabetic group (ST2D) maintained elevated blood glucose levels throughout the experimental period compared to healthy animals.

In male mice (Figure 2A), treatment with EAcFc produced a progressive decrease in glycemia from the second week of treatment, with significant differences observed with respect to the ST2D group at weeks 2, 3 and 4. Towards the end of the study, animals treated with EAcFc had a marked reduction in glucose levels compared to the untreated diabetic group. On the other hand, acarbose (Aca) also showed an antihyperglycemic effect during week 2 at the end of the experimental period. In females (Figure 2B), EAcFc also showed antihyperglycemic activity. Significant reductions in blood glucose were observed from week 1 to week 4 compared to the ST2D group. Compared to acarbose, EAcFc showed a better effect at weeks 2 to 4.

Taken together, these results indicate that EAcFc exerts a subchronic antihyperglycemic effect in mice with streptozotocin-induced type 2 diabetes, with a time-dependent response being observed.

### 2.4. Effects on % Glycated Hemoglobin After Subchronical Administration of Aqueous Extract of Leaves from F. carica

**Figure 3:**
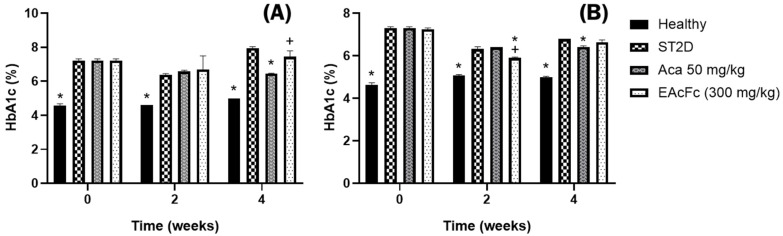
Results of the percentage of glycated hemoglobin values (% HbA1c) in males (**A**) and females (**B**) mice, at baseline (0), 2 and 4 weeks of subchronic analysis. Data are expressed as means ± SEM, *n* = 6. Statistical analysis was performed using two-way ANOVA followed by Tukey’s multiple comparisons test. Differences were considered statistically significant when * *p* < 0.05 comparing the indicated treatment versus the untreated diabetic control group (ST2D), and + *p* < 0.05 comparing the EAcFc group versus the reference drug group (Aca) at the same evaluated week. ST2D: type 2 diabetic group induced by streptozotocin; EAcFc: aqueous extract of *F. carica* leaves; Aca: acarbose.

Figure 3 shows the percentage of glycosylated hemoglobin (% HbA1c) during subchronic treatment with EAcFc in mice with streptozotocin-induced type 2 diabetes mellitus (ST2D). In both sexes, the ST2D group had high HbA1c percentages throughout the experimental period compared to healthy animals, confirming the persistent hyperglycemic state induced by the diabetic model. In males (Figure 3A), treatment with EAcFc did not produce a significant decrease in % HbA1c levels compared to the ST2D group over the course of the study. Although at week 4 a slight trend towards lower values was observed compared to the ST2D group, the percentages of HbA1c remained high and far from those observed in the healthy group. Treatment with acarbose showed a significant reduction in HbA1c at week 4 against ST2D. In females (Figure 3B), the group treated with EAcFc generated a significant decrease in % HbA1c compared to ST2D and Aca at week 2. However, at week 4 the effect of EAcFc did not produce significant differences that would indicate a significant improvement in glycemic control.

Taken together, these results indicate that at the end of the study, treatment with EAcFc was not able to significantly maintain the reversal of the chronic hyperglycemic state associated with type 2 diabetes mellitus, as HbA1c levels remained elevated and close to those of the untreated diabetic group in both sexes.

### 2.5. Effect on Lipid Profile After Subchronical Administration of Aqueous Extract of Leaves from F. carica

The lipid profile of the animals treated during the subchronic trial was evaluated at weeks 2 and 4, in order to determine the effect of EAcFc on metabolic parameters associated with DM2. Serum concentrations of total cholesterol (CHO), triglycerides (TGs), high-density lipoproteins (HDL-c), and low-density lipoproteins (LDL-c) were determined in male and female ST2D mice (Table 1).

In male mice, the ST2D group presented a marked alteration in the lipid profile, mainly characterized by a significant increase in triglyceride and LDL-c levels compared to the healthy group, during the experimental period. Likewise, an increase in all parameters was observed in week 2. Treatment with EAcFc produced a partial improvement in the lipid profile, highlighting a significant reduction in triglycerides at weeks 2 and 4 compared to the ST2D group. Similarly, the extract maintained total cholesterol levels close to those observed in the healthy group during week 2. Regarding HDL-c, EAcFc showed higher values than the ST2D group, while LDL-c levels remained elevated compared to the healthy group. On the other hand, the group treated with acarbose presented high values of CHO, LDL-c and TGs compared to the healthy group, in addition to a less evident reduction in triglycerides compared to the extract.

In females, the ST2D group also showed alterations in lipid metabolism, mainly evidenced by a significant increase in triglycerides and modifications in HDL-c and LDL-c concentrations compared to the healthy group. Treatment with EAcFc significantly decreased triglyceride levels during week 2 compared to the ST2D group; however, around week 4 an increase in this parameter was observed. In relation to total cholesterol, the values remained close to those observed in the healthy group during the study, although a moderate increase was recorded at week 4. In addition, EAcFc maintained LDL-c concentrations similar to those of the healthy group and higher HDL-c levels compared to the diabetic group without treatment. Acarbose showed a similar behavior to the extract on some lipid parameters, although it presented greater increases in LDL-c during both weeks of evaluation.

Taken together, these results suggest that subchronic administration of EAcFc exerts a modulating effect on lipid alterations associated with experimental type 2 diabetes mellitus, particularly on hypertriglyceridemia, also showing sex-dependent differences in the magnitude of the observed metabolic response.

### 2.6. Oral Sucrose and Lactose Tolerance Tests of Aqueous Extract of Ficus carica on Fasted Normoglycemic Mice

To investigate whether the antihyperglycemic effect of EAcFc could be related to the inhibition of a-glucosidase, oral sucrose tolerance (OST) and oral lactose tolerance (OLT) tests were performed, using acarbose as the reference drug.

**Figure 4:**
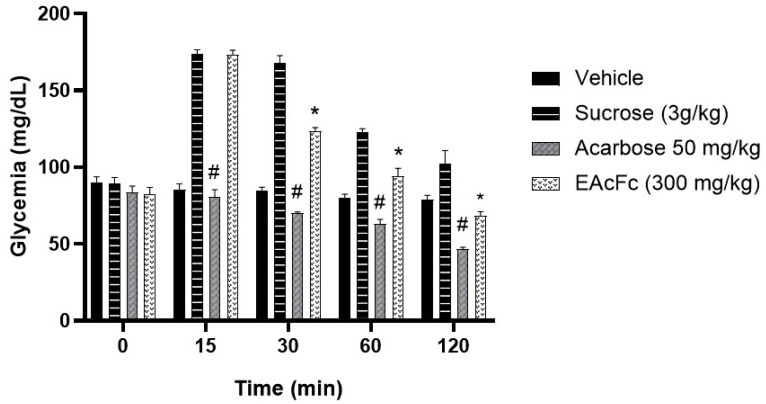
Results of the oral sucrose tolerance test. Data are expressed as the mean ± SEM (*n* = 6). Statistical analysis was performed using two-way ANOVA followed by Tukey’s multiple comparisons test. Differences were considered statistically significant when * *p* < 0.05 comparing the EAcFc + sucrose group versus the sucrose-only control group, and # *p* < 0.05 comparing the Aca + sucrose group versus the sucrose-only control group at the same point. EAcFc was administered at 300 mg/kg; acarbose was administered at 50 mg/kg; sucrose was administered at 3 g/kg.

In the OST test (Figure 4), sucrose administration (3 g/kg) induced a marked increase in glycemia, reaching maximum values at 15 min and remaining elevated up to 120 min compared to the vehicle group. Treatment with acarbose (50 mg/kg) significantly reduced the postprandial hyperglycemic response at 15, 30, 60 and 120 min compared to the sucrose control group. Similarly, treatment with EAcFc (300 mg/kg) produced a significant decrease in glycemia after 30 min and throughout the evaluation period. Although no differences were observed at 15 min compared to the sucrose control group, the extract significantly attenuated the glycemic increase at 30, 60 and 120 min. However, the magnitude of the effect was smaller than that observed with acarbose, whose glycemic values remained consistently lower than those recorded for EAcFc for most of the trial.

**Figure 5:**
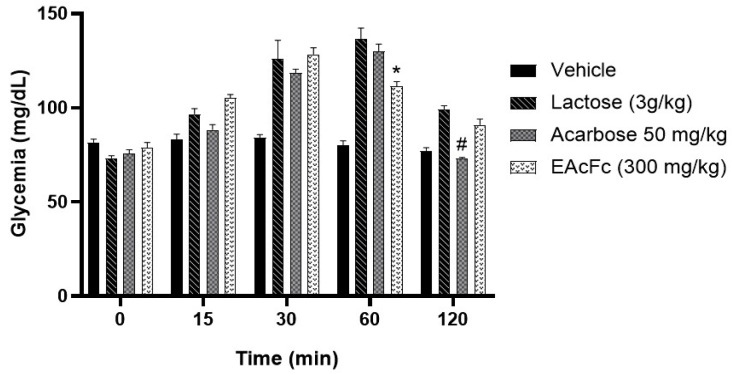
Results of the oral lactose tolerance test. Data are expressed as the mean ± SEM (*n* = 6). Statistical analysis was performed using two-way ANOVA followed by Tukey’s multiple comparisons test. Differences were considered statistically significant when * *p* < 0.05 comparing the EAcFc + lactose group versus the lactose-only control group, and # *p* < 0.05 comparing the Aca + lactose group versus the lactose-only control group at the same point. EAcFc was administered at 300 mg/kg; acarbose was administered at 50 mg/kg; lactose was administered at 3 g/kg.

In the oral lactose tolerance (OLT) test (Figure 5), lactose load produced a significant increase in glycemia, with the hyperglycemic peak being observed at 60 min. Under these experimental conditions, treatment with EAcFc (300 mg/kg) significantly decreased blood glucose levels compared to the lactose-based control group at the same time point, evidencing an attenuation of the postprandial glycemic peak. In contrast, acarbose (50 mg/kg) only showed a significant reduction in glycemia at 120 min.

Taken together, these findings suggest that EAcFc exerts a regulatory effect on the disaccharide-induced postprandial glycemic response, significantly decreasing glycemia during the maximum-elevation phase observed in this experimental model.

### 2.7. Oral Glucose Tolerance Tests of Aqueous Extract of Ficus carica on Fasted Normoglycemic Mice

**Figure 6:**
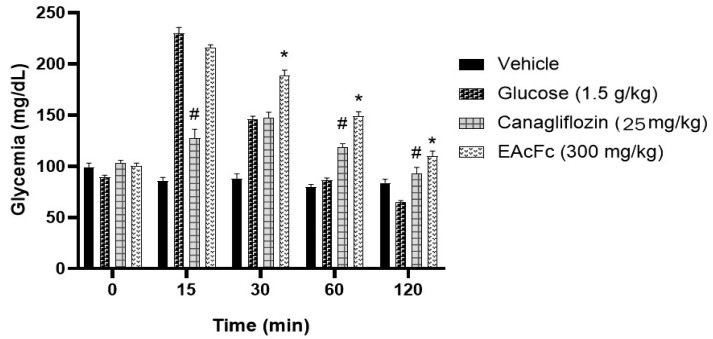
Results of the oral glucose tolerance test. Data are expressed as the mean ± SEM (*n* = 6). Statistical analysis was performed using two-way ANOVA followed by Tukey’s multiple comparisons test. Differences were considered statistically significant when * *p* < 0.05 comparing the EAcFc + glucose group versus the glucose-only control group, and # *p* < 0.05 comparing the canagliflozin + glucose group versus the glucose-only control group at the same point. EAcFc was administered at 300 mg/kg; canagliflozin was administered at 25 mg/kg; glucose was administered at 1.5 g/kg.

To evaluate the possible participation of SGLT-1 in the antihyperglycemic activity of EAcFc, the oral glucose tolerance (OGT) test was performed. The results obtained showed that treatment with EAcFc did not significantly modify the glycemic response induced by oral glucose load compared to the control group throughout the experimental period (Figure 6). In contrast, canagliflozin produced a significant decrease in glycemia at 15 min of evaluation, an effect that was not maintained in subsequent times. These findings indicate that EAcFc does not exert an appreciable inhibitory effect on SGLT-1-mediated intestinal absorption of glucose under the experimental conditions employed.

### 2.8. Isolation and Identification of Secondary Metabolites Present in the Aqueous Extract of Ficus carica Leaves by TLC

**Figure 7:**
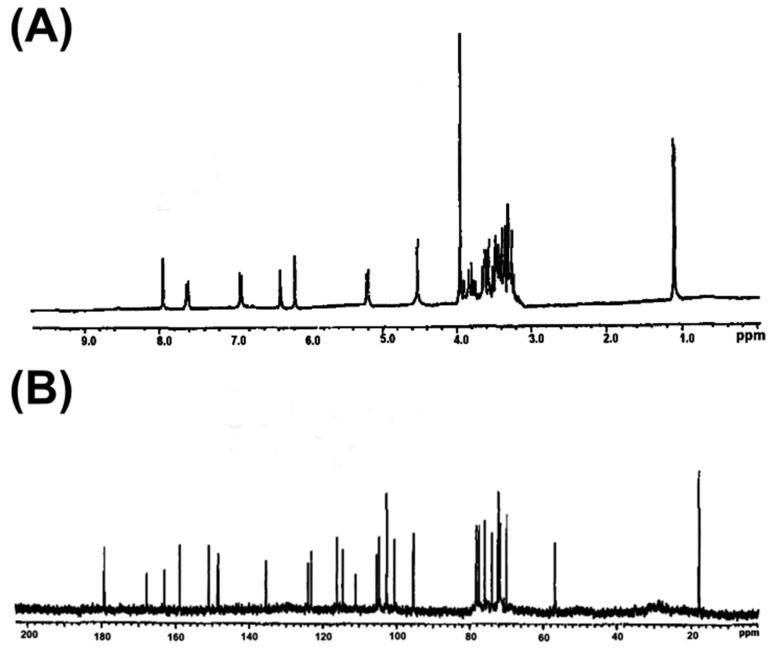
1H-NMR spectra (**A**) and 13C-NMR spectra (**B**) of narcissin isolated from *F. carica* leaves.

**Figure 8:**
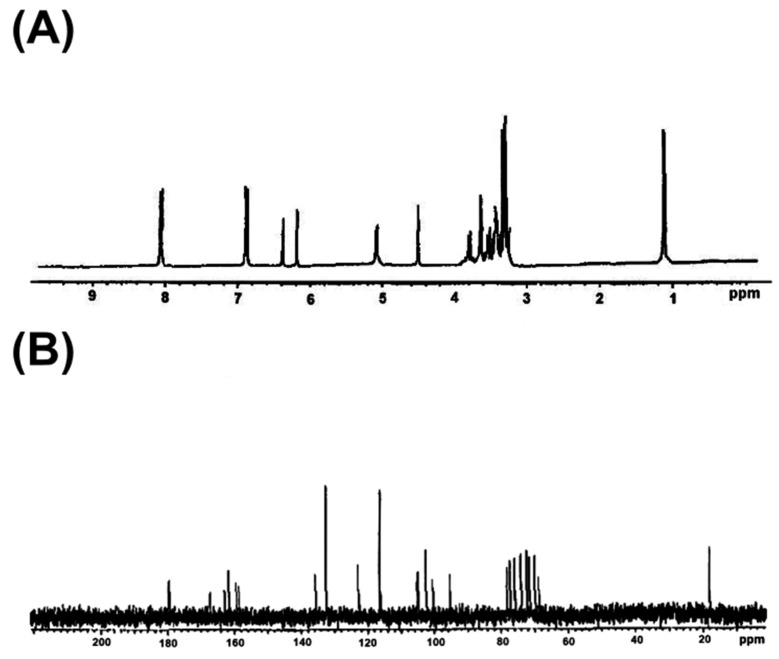
^1^H-NMR spectra (**A**) and ^13^C-NMR spectra (**B**) of nicotiflorin isolated from *F. carica* leaves.

**Figure 9:**
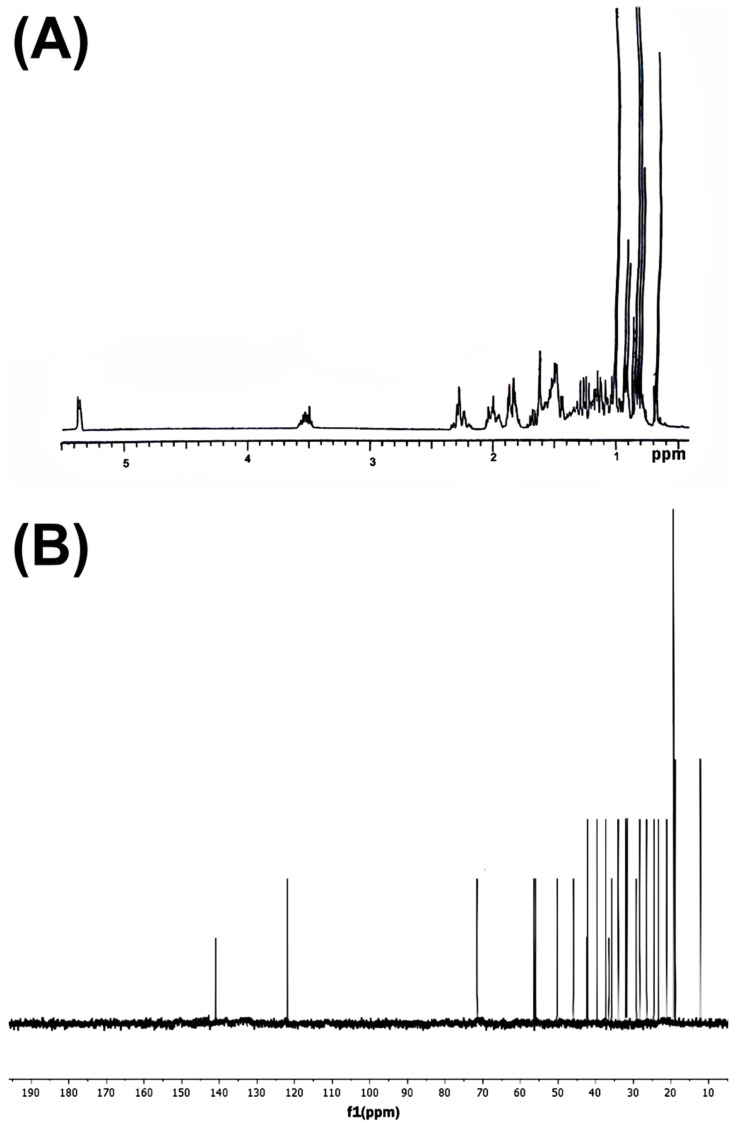
^1^H-NMR spectra (**A**) and ^13^C-NMR spectra (**B**) of β-sitosterol isolated from *F. carica* leaves.

To identify the secondary metabolites, present in EAcFc, a preparative thin-layer chromatography (TLC) on silica gel was performed using the EtOAc:MeOH:H_2_O and Hex:EtOAc elution systems. This procedure allowed the isolation of three compounds (Table 2). Structural elucidation was carried out by analyzing the NMR spectra of ^1^H and ^13^C and comparing them with previously reported data [27,28,29], allowing the identification of compounds such as narcissin (Figure 7), nicotiflorin (Figure 8) and β-sitosterol (Figure 9). The identification of the isolated metabolites was corroborated by comparing them with authentic reference samples available in the laboratory. This phytochemical characterization confirmed the presence of glycosylated flavonoids and a phytosterol as components of the extract.

## 3. Discussion

Type 2 diabetes mellitus (T2DM) continues to represent one of the main public health problems worldwide due to its high prevalence and associated metabolic complications, including persistent hyperglycemia, dyslipidemia, and progressive vascular damage [30]. In middle-income countries, such as Mexico, the burden associated with this disease continues to increase, which has driven the search for new therapeutic alternatives derived from natural products with antihyperglycemic efficacy and a better safety profile. In this sense, several medicinal plant species have aroused interest due to their content of bioactive metabolites with the potential to modulate glycemic and lipid homeostasis [31].

In this context and considering the ethnopharmacological and pharmacological antecedents previously reported for *F. carica*, in the present study the antihyperglycemic activity of the aqueous extract of *F. carica* leaves (EAcFc) was investigated using an experimental model of type 2 diabetes mellitus induced by streptozotocin (ST2D). The evaluation included acute and subchronic studies with the purpose of determining not only its ability to reduce hyperglycemia, but also its effect on metabolic parameters associated with chronic diabetic status, including glycosylated hemoglobin (HbA1c) and lipid profile. Likewise, in order to explore the possible mechanisms involved in the observed activity, oral tolerance tests to different carbohydrates were carried out, aimed at analyzing a possible modulation of the enzyme α-glucosidase, as well as the sodium–glucose cotransporter (SGLT). Finally, a preliminary phytochemical characterization of the extract was carried out by isolating and identifying secondary metabolites, with the aim of relating its chemical composition with the pharmacological effects observed.

Before evaluating the pharmacological activity of the extract, its safety profile was evaluated using the OECD acute toxic class method [26]. The results showed that the oral administration of EAcFc did not cause mortality, behavioral alterations or macroscopic damage to internal organs such as lesions, changes in coloration or an increase or decrease in weight, allowing the extract to be classified within category 5 of acute toxicity (LD_50_ > 3000 mg/kg). These findings suggest a wide margin of safety for the extract under the experimental conditions evaluated. Previous subchronic studies of *F. carica* toxicity support the relative safety of this type of plant preparation [32]. However, although the results obtained suggest low acute toxicity, it is important to consider that chronic toxicological and pharmacokinetic studies are still necessary to more fully establish the safety of the extract after prolonged exposures.

After demonstrating the acute safety of EAcFc, the antihyperglycemic activity of EAcFc was evaluated in mice with streptozotocin-induced type 2 diabetes (ST2D) and nicotinamide. The ST2D model used in this study has been widely used to reproduce metabolic alterations characteristic of DM2, particularly persistent hyperglycemia and partial impairment of pancreatic β cell function [33,34]. Although this model does not fully reproduce the pathophysiological complexity of human type 2 diabetes, it represents a useful tool for evaluating compounds with antihyperglycemic potential.

In the acute assessment, EAcFc showed a moderate ability to lower glycemia in diabetic mice of both sexes. In males, the antihyperglycemic effect was most evident around 24 h after administration, while in females’ significant reductions were observed only in intermediate times. Subsequently, the subchronic effect of the extract was evaluated during four weeks of continuous treatment. The results showed a progressive reduction in glycemia in both sexes, being particularly noticeable in females, where the extract produced significant decreases from the first week and showed a superior effect to that of acarbose during part of the experimental period. In males, although the antihyperglycemic effect was also evident, the response was less pronounced. These results suggest that repeated administration of EAcFc could contribute to improving sustained glycemic control in experimental diabetes. The results obtained are consistent with previous studies carried out with *Ficus carica* leaves, where different types of extracts have shown antihyperglycemic activity in experimental models of streptozotocin-induced diabetes; it has been suggested that these effects have been mainly related to the antioxidant capacity of its phenolic compounds and flavonoids, as well as to its participation in mechanisms involved in glycemic homeostasis and pancreatic function [35]. In particular, a study based on a hydroalcoholic extract of *F. carica leaves*, administered for 28 days, produced a progressive decrease in glycemia, in addition to improving several metabolic parameters related to carbohydrate metabolism in diabetic animals [36]. More recently, in a study based on a dichloromethane extract obtained from *F. carica* leaves, it significantly reduced blood glucose and improved metabolic control in a high-fat, high-streptozotocin-induced diabetes model [37]. It is important to note the chemical distinction based on solvent polarity: while our aqueous extract is rich in highly polar compounds, such as flavonoid glycosides (nacrissin and nicotiflorin), which likely act locally in the gut to inhibit α-glucosidase, non-polar organic solvents like DCM preferentially concentrate lipophilic constituents, such as triterpenes, free aglycones, and fatty acids. This suggests that different active ingredients are at play depending on the preparation method, and the comprehensive antihyperglycemic potential of *F. carica* is multifaceted, involving complementary metabolic pathways. In another study, an ethanolic extract of *F. carica* leaves was shown to decrease glycemia and increase GLUT-2 expression in liver and pancreatic tissue in rats with streptozotocin–nicotinamide-induced diabetes, suggesting a direct involvement of the extract in the regulation of glycemic homeostasis [38]. Taken together, these reports support the antihyperglycemic effects observed in EAcFc and suggest that the metabolites present in *F. carica* leaves could act through multiple mechanisms involved in glucose control.

Despite the observed decrease in blood glucose levels during subchronic treatment, the results corresponding to the percentage of glycosylated hemoglobin (%HbA1c) showed that the extract could not significantly reverse the chronic hyperglycemic state at the end of the study. In males, EAcFc did not produce statistically significant reductions in HbA1c, while in females only a transient decrease was observed during week 2. These findings suggest that, although the extract exerts antihyperglycemic effects on circulating glycemia, its impact on prolonged glycemic control is still limited under the experimental conditions used. HbA1c is one of the most important biomarkers for assessing chronic glycemic control, as it reflects sustained exposure to elevated glucose concentrations. Consequently, the absence of a marked reduction in HbA1c could indicate that the treatment time, the dose used, or the intensity of the antihyperglycemic effect were not sufficient to completely reverse the metabolic alterations induced by the diabetic model. Similar results have been described in experimental studies where improvement in glycemia precedes observable changes in glycosylated hemoglobin, particularly during short treatment periods. Likewise, research carried out with *F. carica* extracts suggests that the effects on HbA1c depend on the intensity and duration of the antihyperglycemic response achieved during treatment [37].

In addition to the effect on glycemia, the impact of EAcFc on the lipid profile was evaluated, considering that dyslipidemia represents one of the main metabolic alterations associated with DM2. In both sexes, diabetic animals presented hypertriglyceridemia and alterations in LDL-c and HDL-c concentrations, confirming the metabolic deterioration induced by streptozotocin. Treatment with EAcFc produced a significant reduction in triglycerides in both sexes during the weeks of study evaluation. In addition, in male mice the extract maintained total cholesterol levels close to those observed in healthy animals. In female mice, the extract maintained LDL-c concentrations like the normoglycemic group and higher HDL-c levels compared to the ST2D group. These results suggest that EAcFc exerts a partial modulatory effect on lipid metabolism associated with experimental diabetes. Previous studies have reported that *F. carica* extracts rich in flavonoids and phenolic compounds can lower triglycerides, improve lipid parameters, and reduce the atherogenic risk index through mechanisms related to antioxidant activity, lipogenic enzyme modulation, and oxidative stress reduction [38,39]. Although molecular markers related to lipid metabolism were not evaluated in the present study, the findings support the possible additional metabolic benefit of the extract on complications associated with DM2.

In order to explore the possible mechanism of action of EAcFc, OST, OLT, OGT tests were performed. The results showed that EAcFc significantly decreased the postprandial hyperglycemic response induced by sucrose and lactose, particularly during periods of maximum glycemic elevation at 30 and 60 min, respectively. These findings suggest a possible partial inhibition of intestinal α-glucosidase enzymes involved in disaccharide hydrolysis. The activity observed was comparable, although to a lesser extent, to that generated by acarbose, a classic α-glucosidase inhibitor used as a pharmacological control.

On the other hand, in the OGT assay, the EAcFc showed no effect on the intestinal absorption of glucose which indicates that the EAcFc probably does not exert an effect on SGLT cotransporter 1, under the experimental conditions employed. These results allow us to infer that the antihyperglycemic mechanism of the extract could be more related to the modulation of the digestion of complex carbohydrates than to the direct inhibition of intestinal glucose transport. Inhibition of these enzymes has been described to delay the release of absorbable monosaccharides and attenuate postprandial glycemic peaks, a mechanism like that exerted by acarbose [40]. This effect could be related to the presence of flavonoids identified in the extract, as various phenolic compounds and flavonoids present in *F. carica* have shown inhibitory activity on digestive enzymes involved in carbohydrate metabolism [19,20,23,35,36]. In contrast, the absence of significant changes during the oral glucose tolerance test indicates that the extract does not significantly interfere with SGLT-1-mediated intestinal absorption of glucose. This interpretation is supported by physiological studies that indicate that inhibition of this transporter generates an evident decrease in the glycemic peak after an oral glucose load, an effect that was not observed in the present study [41]. Taken together, these findings suggest that the antihyperglycemic mechanism of EAcFc would be mainly associated with the modulation of intestinal carbohydrate digestion rather than with a direct inhibition of intestinal glucose transport.

In order to obtain the preliminary phytochemical characterization of EAcFc, a preparative thin-layer chromatography was performed that allowed the isolation of four compounds that were identified by ^13^C and ^1^H NMR data. Three secondary metabolites were identified: narcissin, nicotiflorin and β-sitosterol. This is consistent with what has been reported in other phytochemical studies where the presence of these metabolites has been confirmed in this species [23,35,42]. The presence of these compounds is particularly relevant because several studies have associated glycosylated flavonoids and phytosterols with antihyperglycemic, antioxidant and antihyperlipidemic activities. Nicotiflorin and narcissin, glycosylated flavonols derived from kaempferol and isorhamnetin, respectively, have been associated with various beneficial effects on metabolic homeostasis. These compounds have been described as exerting antioxidant and anti-inflammatory activity and modulating pathways involved in peripheral glucose utilization and the protection of pancreatic β cells. Similarly, they present antiglycant activity by reducing the formation of advanced glycation end products (EFAs), whose accumulation contributes to the development of microvascular and macrovascular complications of diabetes mellitus. In addition, it has been reported that these flavonoids can inhibit the activity of α-glucosidase. This mechanism could contribute to the reduction in postprandial hyperglycemia observed in the present study [43,44]. On the other hand, the c identified in the EAcFc could also contribute significantly to the pharmacological effects observed. Several studies have shown that this phytosterol has antihyperglycemic and lipid-lowering activity in experimental models of streptozotocin-induced diabetes. Proposed mechanisms include stimulating insulin secretion, protecting pancreatic β cells from oxidative damage, improving insulin sensitivity, and regulating lipid metabolism. Likewise, a decrease in serum glucose, glycosylated hemoglobin, CHO and TG has been reported after prolonged administration [45], effects that partially coincide with the metabolic modifications observed in the present study.

Overall, the results obtained suggest that the antihyperglycemic and lipid profile modulating activity observed for EAcFc could be related, at least partially, to the combined presence of these secondary metabolites. However, because the extract constitutes a complex mixture of bioactive compounds, the participation of synergistic effects between flavonoids, sterols and other minority constituents not identified in this study cannot be ruled out.

Despite these promising findings, several unanswered questions remain that warrant further investigation. First, the precise reason for the lack of significant reduction in HbA1c levels, despite notable improvement in fasting glycemia, is not fully elucidated; this suggests that longer-term subchronic or chronic studies exceeding four weeks are required to observe sustained changes in red blood cell glycation. Second, while the attenuation of the postprandial hyperglycemia strongly suggests intestinal α-glucosidase inhibition, the direct enzymatic interaction and potential effects on other critical metabolic pathways, such as insulin resistance and peripheral glucose transporter expression (e.g., GLUT-4), remain to be determined. Furthermore, the exact synergistic contribution of the isolated metabolites versus other unidentified minor constituents in the complex aqueous matrix still unknown.

Therefore, future perspectives should focus on comprehensive mechanistic and phytochemical profiling. While the NMR analysis utilized in this study provided definitive structural confirmation for the isolated pure compounds, its reproducibility when applied to complex plant extracts or isolated fractions is often limited by low sensitivity and vulnerability to signal overlap from trace co-eluting impurities. To overcome these limitations and assess batch-to-batch repeatability, high-resolution chromatographic techniques, such as HPLC-MS/MS, are essential. LC-MS not only offers superior sensitivity and dynamic separation of complex mixtures but also allows for the robust quantification necessary to establish a standardized phytochemical fingerprint of the extract. Additionally, as the stability of natural products can impact long-term efficacy, future studies must incorporate time-course analytical evaluations (e.g., comparative NMR and HPLC tracking over several weeks) to rule out the degradation of active metabolites during prolonged storage of treatment. Furthermore, future research should incorporate in silico approaches, such as molecular docking targeting specific crystallographic structures of α-glucosidase, to provide deep structural insights into the binding affinities and inhibitory mechanisms of narcissin, nicotiflorin, and β-sitosterol. These computational approaches must be complemented with direct in vitro enzymatic assays, pharmacokinetic tracking of the active metabolites, and long-term in vivo chronic toxicity studies to fully validate the therapeutic potential and clinical safety of *F. carica* as an adjunctive antidiabetic agent.

## 4. Materials and Methods

### 4.1. Reagents, Drugs, and Chemicals

All the reagents used were of analytical grade. Nicotinamide (99.5%, PN: 47865-U), streptozotocin (75% α-anomer, PN: S0130-5G), acarbose (PN: PHR1253-500 mg), canagliflozin (95%, PN: 721174-1 G), glucose (PN: D9434-1 kg), sucrose (99.5% GC, PN: S9378-1 kg) and lactose (99%, PN: 61339-25 g) were purchased from Sigma-Aldrich (St. Louis, MO, USA). Chromatographic separations were performed using pre-coated silica gel 60 F254 plates (Merck, Darmstadt, Germany).

### 4.2. Plant Material

**Figure 10:**
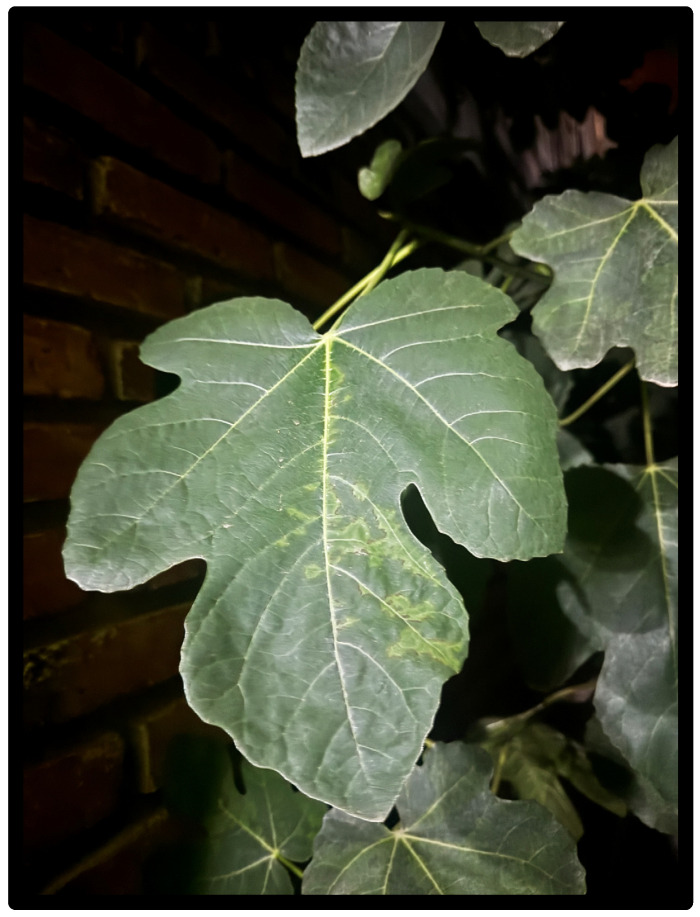
Leaf from *Ficus carica* L.

*Ficus carica* L. leaves (Figure 10) were collected in Col Cuitláhuac, Alcaldía Tláhuac in Mexico City (19°16′ N, 99°00′ W), by Dr. Fernando Calzada Bermejo, Senior Researcher of the Medical Research Unit in Pharmacology (UIMF) at the Mexican Institute of Social Security (IMSS) of the Siglo XXI National Medical Center (CMN XXI).

The botanical identification of the plant material was carried out by Santiago Xolalpa at the herbarium IMSSM of the Instituto Mexicano del Seguro Social, Centro Médico Nacional Siglo XXI. A voucher specimen was deposited under reference number 17010.

### 4.3. Preparation of the Aqueous Extract of the Leaves from Ficus carica L.

Collected *Ficus carica* leaves were thoroughly washed with distilled water, shade-dried at room temperature to preserve thermolabile metabolites, and pulverized into a fine, homogeneous powder using a mechanical grinder. To prepare the aqueous extract (EAcFc) mimicking traditional infusion methods, 1.5 g of the powdered leaves were placed in filter paper sachets and extracted with 125 mL of purified boiling water (100 °C) for exactly 20 min under continuous stirring. After extraction, the mixture was filtered through Whatman No. 1 filter paper to completely remove plant debris. The resulting aqueous filtrate was concentrated under reduced pressure at 40 °C using a rotary evaporator (Büchi Labortechnik AG, Flawil, Switzerland). The resulting residue was dried to a constant mass, yielding 355.74 mg of dry extract (yield: 23.7% *w*/*w*). The EAcFc was stored at 4 °C in airtight, light-protected containers until further use in biological and phytochemical assays.

### 4.4. Isolation, Extraction and Identification of Flavonoids

**Figure 11:**
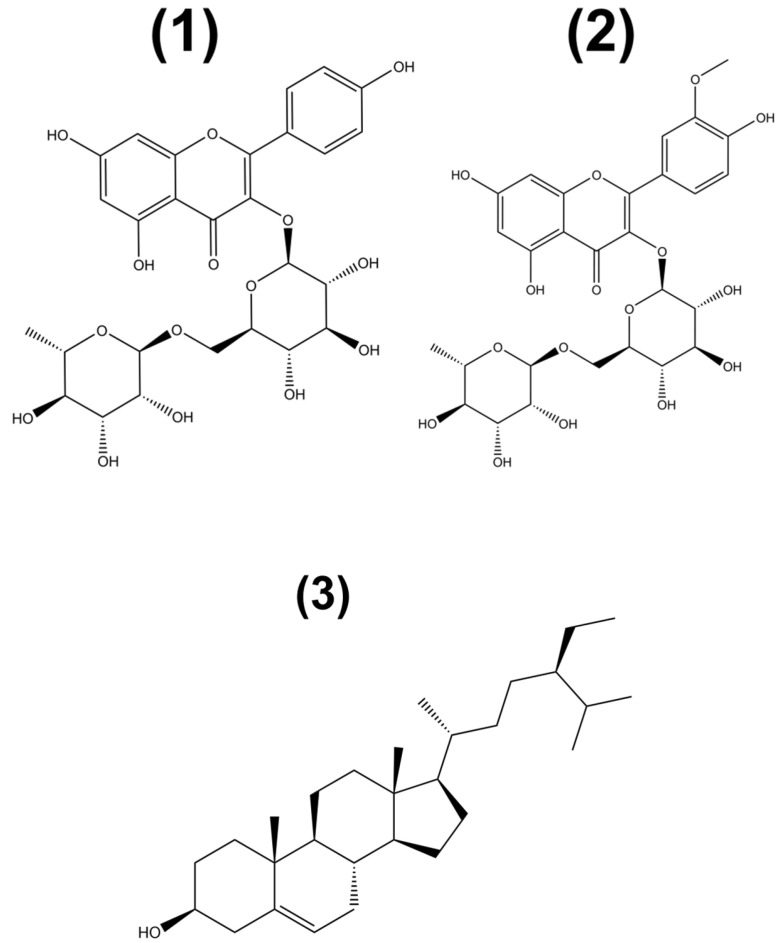
Structure of secondary metabolites isolated from *Ficus carica* L: Nicotiflorin (**1**), narcissin (**2**), and *b*-sitosterol (**3**).

The isolation and purification of flavonoids was performed by preparative thin-layer chromatography (Merck 60F-254 silica gel). A portion (100 mg) was purified using a mixture of EtOAc:MeOH:water (10:1,6:1,3, *v*:*v*) to obtain two flavonoid glycosides, nicotiflorin (**1**) and narcissin (**2**); the process was repeated as needed. For the isolation of phytosterol, β-sitosterol (**3**) (Figure 11), the Hex:AcOEt system (8:2, *v*:*v*) was used as a mobile phase.

The preparative TLC allowed the isolation of three compounds that were identified by reported ^13^C and ^1^H NMR data as the flavonoids, narcissin (Figure 7) and nicotiflorin (Figure 8) and a phytosterol, β-sitosterol (Figure 9).

### 4.5. In Vivo Assays

#### 4.5.1. Animals

Male and female BALB/c mice were obtained aged between eight and ten weeks, weighing 20 ± 5 g, at the Veterinary Clinic of the National Medical Center “Siglo XXI” of the Mexican Institute of Social Security (IMSS, Mexico City, Mexico). The animals were housed under controlled environmental conditions, including a temperature of 22 ± 2 °C, relative humidity of approximately 50% and a photoperiod of 12 h of light and 12 h of darkness. Throughout the experimental period, mice had free access to purified water and a standard laboratory diet for rodents (LabDiet^®^ 5001, St. Louis, MO, USA). All experimental protocols were reviewed and approved by the Research and Ethics Committee of the Specialized Hospital of the National Medical Center “Siglo XXI” (IMSS; approval number R-2025-3601-366). Animal handling and experimental procedures were carried out in accordance with applicable Mexican regulations governing the care and use of laboratory animals and followed internationally accepted principles for animal welfare in biomedical research [46].

#### 4.5.2. Experimental Design, Randomization, and Replication

For all in vivo efficacy experiments, the sample size consisted of *n* = 6 independent animals per group, representing biological replicates. To minimize selection bias, animals were randomly assigned to their respective experimental groups using a computer-generated randomization sequence created with GraphPad Prism software (version 8.2.1; GraphPad Software, San Diego, CA, USA). In the diabetic models, this randomization was stratified based on baseline fasting blood glucose prior to treatment. Furthermore, to ensure analytical reproducibility, all biochemical measurements (including blood glucose readings, %HbA1c, and lipid profile parameters) were performed using technical replicates (duplicate or triplicate readings) for each biological sample.

#### 4.5.3. Acute Oral Toxicity

Acute oral toxicity was assessed according to the Organization for Economic Cooperation and Development (OECD) Guideline 423 (Acute Oral Toxicity—Acute Toxic Class Method) for the testing of chemicals [26]. Female Balb/c mice were randomly allocated into three experimental groups (*n* = 3 per group). Following an overnight fasting period, animals received a single oral administration via intragastric gavage while maintaining free access to water throughout the study. The experimental groups consisted of a healthy control group (untreated), a vehicle control group receiving 2% Tween 80 in water, and a treatment group administered the aqueous stem extract at a dose of 3000 mg/kg body weight. After dosing, animals were continuously observed during the first 4 h for the detection of clinical signs of toxicity, including lethargy, sedation, tremors, convulsions, diarrhea, behavioral abnormalities, and mortality. Subsequently, animals were monitored daily for a total observation period of 14 days.

At the end of the experimental period, all animals were slaughtered and subjected to a pathological examination. Major organs, including the liver, stomach, intestines, spleen, and kidneys, were carefully inspected for macroscopic alterations potentially associated with treatment-related toxicity. Organ weights were also recorded and compared with those of the control group.

Based on the mortality and toxicity findings, the median lethal dose (LD_50_) was estimated and the extract was classified according to the OECD acute toxicity classification system as follows: Category 1 (highly toxic, ≤5 mg/kg), Category 2 (toxic, >5–50 mg/kg), Category 3 (harmful, >50–300 mg/kg), Category 4 (low toxicity, >300–2000 mg/kg), and Category 5 (LD_50_ > 2000 mg/kg, indicating relatively low acute toxicity).

#### 4.5.4. Experimental Type 2 Diabetes Induction

Experimental type 2 diabetes mellitus (ST2D) was induced using a streptozotocin–nicotinamide model, as previously described [47]. Before diabetes induction, mice fasted for 16 h with free access to water. Streptozotocin (STZ) was prepared in a 0.1 M citrate cold buffer (pH 4.0) and administered intraperitoneally at a dose of 100 mg/kg on days 1 and 3. Nicotinamide (NA) was dissolved in sterile saline and administered intraperitoneally at 240 mg/kg, 30 min after the first STZ injection on Day 1. Following the induction protocol, animals received a 10% sucrose solution ad libitum for 48 h to minimize the risk of acute hypoglycemia associated with STZ administration. Thereafter, the sucrose solution was replaced with purified drinking water.

Blood glucose levels were determined 72 h after the final STZ administration using blood samples collected from the tail vein. Glycemia was measured by the glucose oxidase method using reactive test strips and a portable glucometer (Accu-Chek^®^ Performa Blood Glucose System, Roche Diagnostics, Basel, Switzerland). Animals exhibiting fasting blood glucose concentrations between 290 and 390 mg/dL were considered diabetic and were subsequently included in the experimental study.

To verify the preservation of residual pancreatic β-cell function, characteristic of the STZ–NA-induced T2DM model, diabetic mice received a single oral dose of glibenclamide (5 mg/kg). The hypoglycemic response to this insulin secretagogue was used as an indirect indicator of functional β-cell survival following partial protection conferred by nicotinamide against STZ-induced pancreatic damage [48].

### 4.6. Acute Evaluation of Aqueous Extracts from Ficus carica

Only animals with fasting glucose levels between 290 and 390 mg/dL and a positive hypoglycemic response to glibenclamide were considered for the study. After confirming the diabetic status, the animals were randomly distributed into four experimental groups (*n* = 6 per group) for each sex: (i) normoglycemic control group, composed of healthy animals that received the vehicle (preadolescents 80 to 2% in water); (ii) diabetic control group (ST2D), composed of diabetic animals treated only with the vehicle; (iii) the reference group, composed of diabetic animals treated with acarbose (50 mg/kg); and (iv) group treated with aqueous extract of *Ficus carica* leaves (EAcFc) at a dose of 300 mg/kg.

Blood samples were collected from the tail vein, and glucose concentrations were determined using a portable glucometer (Accu-Chek^®^ Performa Blood Glucose System, Roche Diagnostics, Basel, Switzerland). Glycemic measurements were performed immediately before treatment administration (time 0) and at 0.5, 1, 3, 5, and 7 h post-treatment. The antihyperglycemic activity of EAcFc was evaluated by comparing the temporal changes in blood glucose concentrations with those observed in the corresponding control groups.

### 4.7. Subchronic Evaluation of Aqueous Leaf Extract of Ficus carica in the ST2D Mice Model

Following acute antihyperglycemic evaluation, the same diabetic animals were included in a subchronic study designed to evaluate the sustained metabolic effects of the aqueous extract. Treatments were administered orally once daily for 4 weeks. Blood glucose concentrations were measured weekly using a portable glucometer (Accu-Chek^®^ Performa Blood Glucose System, Roche Diagnostics, Basel, Switzerland) to monitor changes in glycemic status over time.

To further investigate the impact of treatment on chronic glycemic control and diabetes-related dyslipidemia, glycated hemoglobin (HbA1c) and serum lipid profile parameters (total cholesterol, triglycerides, HDL-c, and LDL-c) were evaluated at baseline and at weeks 2 and 4 of treatment.

#### 4.7.1. Measurement of % HbA1c

Glycated hemoglobin (HbA1c) was measured as an indicator of long-term glycemic control. Whole-blood samples were obtained from the tail vein of treated animals and analyzed using a boronate affinity chromatography-based automated system (Clover HbA1c Analyzer, Infopia Co., Ltd., Anyang, Republic of Korea). HbA1c values were determined following the manufacturer’s recommendations and expressed as the percentage of glycated hemoglobin relative to total hemoglobin (%HbA1c).

#### 4.7.2. Lipid Profile Measurement

For lipid profile determination, blood samples were collected from the tail vein of the animals at the designated experimental time points. Serum lipid parameters were measured using a VERI-Q^®^ monitoring system (MiCoBioMed Co., Ltd., Seongnam, Republic of Korea), following the manufacturer’s instructions. The analyzed parameters included total cholesterol (TC), triglycerides (TGs), high-density lipoprotein cholesterol (HDL-c), and low-density lipoprotein cholesterol (LDL-c).

### 4.8. Oral Sucrose and Lactose Tolerance Test of Aqueous Leaf Extract of Ficus carica in Fasted Normoglycemic Mice

The possible involvement of aqueous extract of *Ficus carica leaves* (EAcFc) in modulating intestinal glucosidase activity was investigated using oral sucrose tolerance (OSTT) and oral lactose tolerance (OLTT) tests. Male and female Balb/c mice subjected to a night fast and randomly assigned to experimental groups (*n* = 6 per group). The animals received a vehicle (2% Tween 80 in distilled water) or EAcFc (300 mg/kg); the animals that received the disaccharide load (lactose or sucrose only) or acarbose (50 mg/kg) were included as a positive control due to the well-established α-glucosidase inhibitory activity. All treatments were administered orally through an esophageal cannula. Thirty minutes later, the mice were challenged with an oral carbohydrate load composed of sucrose or lactose (3 g/kg body weight). Blood samples were collected from the caudal vein immediately prior to carbohydrate administration 0, 15, 30, 60, and 120 min. Blood glucose concentrations were determined using a portable glucometer (ACCU-CHEK^®^ Performa, Roche Diagnostics, Basel, Switzerland). Changes in postprandial glycemia were then analyzed to assess the ability of EAcFc to attenuate carbohydrate-induced glucose excursions, indicating a possible inhibitory effect on intestinal activity of α-glucosidase.

### 4.9. Oral Glucose Tolerance Assay of Aqueous Leaf Extract of Ficus carica in Fasted Normoglycemic Mice

To evaluate the potential effect of EAcFc on intestinal glucose handling, an oral glucose tolerance test (OGTT) was conducted following the protocol described above for the carbohydrate tolerance assays. After an overnight fast, mice received the corresponding treatments by oral gavage. Canagliflozin (50 mg/kg) was used as the pharmacological reference, whereas vehicle-treated animals served as controls. Thirty minutes later, an oral glucose load (1.5 g/kg) was administered. Blood glucose levels were determined from tail vein samples collected at baseline and after glucose administration using a glucometer (ACCU-CHEK^®^ Performa, Roche Diagnostics, Basel, Switzerland). The same sampling intervals and analytical procedures employed in the OSTT and OLTT were maintained throughout the experiment.

### 4.10. Statistical Analysis

Results are expressed as mean ± standard error of the mean (SEM). Statistical analyses were performed using GraphPad Prism version 8.2.1 (GraphPad Software, San Diego, CA, USA). For experiments involving repeated measurements over time, data were analyzed using two-way analysis of variance (ANOVA) followed by Tukey’s multiple-comparison test. Single-time-point comparisons were evaluated using one-way ANOVA followed by the same post hoc procedure. Differences were considered statistically significant when *p* ≤ 0.05.

### 4.11. Graphical Abstract

The graphical abstract was conceptualized by the authors and refined using the AI tool Gemini 3.1 Pro (Google, Mountain View, CA, USA). The AI was utilized to optimize the visual layout and organizational structure of the biological mechanisms depicted, ensuring clarity and adherence to the journal’s visual requirements. All scientific content, chemical structures, and biological pathways were verified by the authors to ensure accuracy.

## 5. Conclusions

The results of the present study demonstrate that the aqueous extract of *Ficus carica* leaves (EAcFc) has antihyperglycemic activity in an experimental model of type 2 diabetes mellitus induced by streptozotocin and nicotinamide. Acute and subchronic treatment significantly reduced blood glucose levels, with the effect being most evident during prolonged administration. In addition, the extract showed a modulating action on some alterations in the lipid profile associated with diabetic status, particularly on triglyceride levels.

Oral carbohydrate tolerance assays suggest that its mechanism of action could be at least partially related to the inhibition of intestinal α-glucosidases, while no evidence of significant involvement of the SGLT-1 transporter was observed. Likewise, the identification of narcissin778–779, nicotiflorin and β-sitosterol provides a phytochemical basis that could help explain the pharmacological effects observed. However, the absence of a sustained reduction in HbA1c indicates that the extract failed to significantly reverse the chronic hyperglycemic state under the conditions evaluated. Taken together, these findings support the potential of EAcFc as a source of bioactive compounds with antihyperglycemic and lipid metabolism-modulating activity, although further studies are required to elucidate its mechanisms of action and long-term therapeutic potential.

## Figures and Tables

**Table 1:** Lipid profile values (cholesterol, triglycerides, HDL-c, and LDL-c) at 2 and 4 weeks of subchronical assay.

**Lipid Profile Male**				
Cholesterol (mg/dL)			Triglycerides (mg/dL)	
Treatment	Week		Week	
	2	4	2	4
Healthy	102.5 ± 6.3	121.2 ± 0.9	42.7 ± 1.5	44 ± 1.6
ST2D	145.3 ± 21 ^#^	120.2 ± 0.5	158 ± 17.5 ^#^	156 ± 19.4 ^#^*
Aca	171 ± 0.5 ^#^*	171.2 ± 1.5 ^#^*	136.2 ± 1.5 ^#^*	133.2 ± 2 ^#^*
EAcFc	100 ± 1.41 *	117.8 ± 12 *	62.2 ± 12 *	89.7 ± 3.3 ^#^*
HDL-c (mg/dL)			LDL-c (mg/dL)	
Treatment	Week		Week	
	2	4	2	4
Healthy	36.5 ± 1	39.5 ± 1	28.2 ± 0.5	52.5 ± 1
ST2D	86.6 ± 4 ^#^	37.5 ± 9.6	61 ± 4.6 ^#^	60.2 ± 3.9 ^#^
Aca	83 ± 2 ^#^	52.2 ± 5.1 ^#^*	61 ± 0.3 ^#^	60.2 ± 0.9 ^#^
EAcFc	77.5 ± 10.2 ^#^	65.6 ± 5.3 ^#^*	59 ± 1.4 ^#^	59.7 ± 1.2 ^#^
**Lipid Profile Female**				
Cholesterol (mg/dL)			Triglycerides (mg/dL)	
Treatment	Week		Week	
	2	4	2	4
Healthy	98.6 ± 1.4	106.2 ± 6.6	41.5 ± 1	41 ± 1.2
ST2D	99.2 ± 0.5	99 ± 0.8	138 ± 2 ^#^	130.7 ± 5.5 ^#^
Aca	98.7 ± 0.5	105.5 ± 0.5	109.2 ± 0.95 ^#^*	110.2 ± 1.7 ^#^*
EAcFc	100 ± 1.4	112.7 ± 17 *	77 ± 16 ^#^*	119 ± 4 ^#^
HDL-c (mg/dL)			LDL-c (mg/dL)	
Treatment	Week		Week	
	2	4	2	4
Healthy	35.7 ± 0.5	39.7 ± 1	27.7 ± 0.5	32.7 ± 1.5
ST2D	33.5 ± 1	50.7 ± 0.5	61.7 ± 0.5 ^#^*	25.2 ± 0.5 ^#^*
Aca	98.5 ± 1 ^#^*	53.7 ± 0.9	76 ± 1.1 ^#^*	77.3 ± 1.6 ^#^*
EAcFc	73.5 ± 13 ^#^*	62.5 ± 19 *	32 ± 0.8 ^#^*	31.5 ± 2 *

Results expressed as the mean ± SEM, *n* = 6. # *p* < 0.05 vs. healthy control; * *p* < 0.05 vs. ST2D control. ST2D: type 2 diabetic group induced by streptozotocin; EAcFc: aqueous extract of *F. carica* leaves; Aca: acarbose.

**Table 2:** Retention factor, name, molecular weight, and molecular formula of compounds present in the aqueous extract of leaves from *F. carica*.

Compound Name	R.f. *	Molecular Weight (g/mol)	Molecular Formula
Narcissin	0.34	624.5	C_28_H_32_0_16_
Nicotiflorin	0.36	594.5	C_27_H_30_0_15_
*β*-sitosterol	0.42	414.70	C_29_H_50_0

* R.f.: Retention factor.

## Data Availability

The original contribution presented in this study is included in the article. Further inquiries can be directed to the corresponding authors.
